# Different clinicopathological features between young and older patients with pulmonary adenocarcinoma and ground-glass opacity

**DOI:** 10.1038/s41598-024-66910-4

**Published:** 2024-07-08

**Authors:** Xingbing Lu, Yuzuo Chen, Yuxiao Li, Mengli Tang, Xi Zheng

**Affiliations:** 1 Department of Laboratory Medicine, West China Hospital, Sichuan University, Chengdu, China; 2 West China Hospital, Sichuan University, Chengdu, China; 3 Lung Cancer Center, West China Hospital, Sichuan University, Guoxue Lane 37, Chengdu, Sichuan Province China; 4 Department of Thoracic Surgery, West China Hospital, Sichuan University, Chengdu, China

**Keywords:** Pulmonary ground-glass opacity (GGO), Pulmonary adenocarcinoma, Clinical characteristics, Cancer, Cancer

## Abstract

After the recommendation of computed tomography as a routine procedure for lung cancer screening, an increasing number of young adults have been diagnosed with pulmonary ground-glass opacity (GGO). Up to 63% of pulmonary nodules with a GGO component can be malignant. Since young cancer patients have limited exposure to environmental mutagens, they have special characteristics and needs. This study sought to compare the clinicopathological characteristics of young and old patients with GGO-associated lung adenocarcinoma (GGO-LUAD). Clinicopathological data from 203 patients who underwent video-assisted thoracoscopic surgery between January 2018 and April 2020 for pulmonary GGO component nodules were reviewed. Lung nonmucinous adenocarcinoma patients younger than 40 years old and older than 40 years old were enrolled: 103 patients ≤ 40 years old and 100 patients > 40 years old. The relevant clinicopathological features, including sex, smoking status, tumor size, pathological characteristics, radiographic features and prognosis of pulmonary nodules, were evaluated. Univariate analyses were applied for comparisons between groups. The differences in baseline characteristics (sex, smoking status, tumor location) between the different age groups were not significant. Young patients were more likely to have tumors < 1 cm in size, while older patients predominantly had tumors > 2 cm in size. The mean percentage of invasive adenocarcinoma was greater in the elderly group. Young and older patients seemed to have similar subtypes of adenocarcinoma (*p* > 0.05) but had different degrees of differentiation (*p* < 0.001). The 3-year overall survival (OS) and recurrence-free survival (RFS) of the young group were 100% and 99.03%, respectively, while the 3-years OS and RFS of the older group were 99% and 98%, respectively. Our work revealed that young patients with malignant pulmonary nodules and GGOs have distinct pathological subtypes. Patients with GGOs of different ages have different clinicopathological characteristics. The 3-year prognosis of young patients with malignant pulmonary nodules with GGOs is satisfactory.

## Introduction

Lung cancer is the predominant cause of cancer-related mortality worldwide^1^. China has reported a significant incidence of lung cancer, with 815,000 new cases in 2020, along with high mortality rates^2^. The primary treatment for early stage of lung cancer is surgery. For patients diagnosed in advanced or metastasis stage, the options of treatment are determined based on many factors, like the site of tumor and the patient’s performance status^3^. And now immune checkpoint inhibitors (ICIs) have become one of a best first-line treatment for lung cancer^4^. However, ICIs are associated with higher risk of liver toxicity, which means that it’s important to monitor liver function^5^. What’s more, prognostic biomarkers like albumin levels are needed to help the decision-making process with ICIs^6^.

A large proportion of patients with lung cancer are diagnosed at an intermediate or late stage, and the 5-years relative survival rate is only 15%^7,8^^.^ In contrast, the 5-years survival rate for patients diagnosed with stage 1A disease (early stage) is over 75%^9^. Thus, diagnosing lung cancer at earlier stages is critical for improving patient outcomes. Low-dose computed tomography (LDCT) and high-resolution computed tomography (HRCT) are common methods for diagnosing early-stage lung cancer^10–13^. Although LDCT can reduce radiation, HRCT is still the first-choice imaging modality for diagnosing ground-glass opacity (GGO) due to its higher resolution^14^.

Previously, older adults were the primary population for lung cancer screening, and young people under the age of 40 rarely had lung cancer, with an incidence of only 0.6–4.6%^15–17^. However, with the widespread adoption of LDCT and the improved resolution of LDCT imaging, the incidence of early-stage lung cancer, characterized by GGO as a radiological feature, has been steadily increasing in routine clinical practice^18,19^. While GGOs, such as focal interstitial fibrosis, inflammation, and hemorrhage, are not always indicative of malignancy^20^, persistent GGOs may indicate the presence of various subtypes of lung adenocarcinoma (LUAD), including adenocarcinoma in situ (AIS), minimally invasive adenocarcinoma (MIA), and invasive adenocarcinoma (IAC)^20,21^. Therefore, the diagnosis and treatment of young patients with GGO-associated LUAD (GGO-LUAD) have become a focus of clinical attention. Unlike pure solid-LUAD, GGO-LUAD is characterized by GGO and has a good prognosis^22^. Several studies have follwoed young GGO-LUAD patients who underwent video-assisted thoracoscopic surgery (VATS), most of whom underwent VATS at an early stage. The results revealed a favorable postoperative prognosis, with 100% recurrence-free survival (RFS) and overall survival (OS) at 3 years^23,24^^.^ However, there are few reports on the clinicopathological characteristics and prognosis of young patients with GGO-LUAD^23^. Due to their limited exposure to environmental mutagens, young cancer patients exhibit distinct characteristics and needs compared to older patients, such as different tumor types and TNM stages^25^. Therefore, it is necessary to compare the clinicopathological characteristics of young and older patients with those of patients with GGO-LUAD.

In this study, we reviewed the clinical data of patients who underwent VATS for a diagnosis of GGO-LUAD from March 2018 to April 2020. The patients were divided into younger (≤ 40 years) and older groups (> 40 years) according to their age. Our study aimed to evaluate and compare the clinicopathological characteristics and prognoses of patients in both groups.

## Materials and methods

### Patients

The clinical data of 203 patients who underwent VATS and were diagnosed with LUAD between January 2018 and April 2020 due to pulmonary GGO component nodules was reviewed. The inclusion criteria were as follows: (1) patients with pulmonary adenocarcinoma with a GGO-containing component confirmed on a thin-section computed tomography (CT) scan and postoperative pathology confirmed as nonmucinous adenocarcinoma of the lung; (2) no other tumors; (3) mediastinal lymph nodes evaluated by enhanced CT of the chest before surgery, with no significantly enlarged mediastinal lymph nodes; (4) no distant metastasis; and (5) complete R0 resection. The exclusion criteria were as follows: (1) postoperative pathology was benign; (2) preoperative examination revealed mediastinal lymph node metastasis; and (3) postoperative pathology confirmed nonadenocarcinoma (mucinous adenocarcinoma, squamous, large cell, small cell, etc.). Relevant clinicopathological features, including sex, smoking status, TNM stage, pathological characteristics, radiographic features, and the prognosis of pulmonary nodules, were evaluated. Univariate analyses were applied to compare groups. Each patient signed an informed consent form before the study. This retrospective study was approved by the Ethics Committee of West China Hospital, Sichuan University. The study followed the Declaration of Helsinki (as revised in 2013), a set of ethical principles to protect participants and draw reliable conclusions^26^.

### Radiological and pathological evaluation

All lesions were evaluated using HRCT images of the GGO nodes. All chest CT scans were performed during complete inspiration, and lesions, particularly GGO nodules, were retrospectively examined. Tumor diameter was defined as the maximum axial diameter of the nodule at the lung window. A solid lesion was defined as a homogeneous increase in lung parenchymal density that obscured the airway wall and vascular margins. In contrast, a GGO was defined as increased lung turbidity preserving the bronchial and vascular margins. The preoperative CT scan of each lesion was reviewed blindly by two experienced radiologists.

### Histologic evaluation

All clinical specimens were examined and recorded by two pathology specialists. The pathology subtype, size, location, differentiation, TNM stage, visceral pleural invasion, vascular tumor thrombus, lymphovascular invasion, and spread through the airspace were reviewed for each nodule according to the International Association for the Study of Lung Cancer (IASLC)/American Thoracic Society (ATS)/European Respiratory Society (ERS) guidelines.

### Surgical approach

The patient was placed in the lateral position, and double-lumen tracheal intubation was performed. Thoracoscopy was used to detect adhesions in the thoracic cavity. The specific location of the nodule was determined by finger exploration or preoperative CT-guided hook-wire localization. The lesions were resected using cutting staplers at least 2 cm from the edge of the nodules. Based on rapid pathological results and comprehensive consideration of the patient’s lung function , futher surgical treatments such as wedge resection, segmentectomy, lobectomy, or radical resection were guided.

### Follow-up

Patients were routinely followed up after surgery by telephone interview or clinic visit until September 30, 2023. In the first 2 years after surgery, physical examinations, tumor marker data, abdominal ultrasound and chest CT data were reviewed every 6 months; these indicators were reviewed annually after 3 years. Bone electroconvulsive therapy (ECT), brain magnetic resonance imaging (MRI), or positron emission tomography-CT (PET-CT) were performed only if recurrence or metastasis were suspected. RFS was defined as the time from surgery until the first recurrence or the last follow-up. Overall survival (OS) was defined as the time from surgery until death from any cause or the last follow-up.

### Statistical analysis

The measured data are expressed as the mean ± standard deviation, and t-tests analyzed differences between groups. Categorical variables are summarized by number (proportion) and were compared with the chi-square test or Fisher’s exact test. OS and RFS curves were plotted using the Kaplan–Meier method and compared with the log-rank test. *p* < 0.05 was considered statistically significant. All data were analyzed using Statistical Package Social Sciences 22.0 and GraphPad Prism software version 8.0.

## Results

### Clinical characteristics

A total of 203 patients were included in the study, with103 patients aged ≤ 40 years and 100 patients aged > 40 years. Table 1 summarizes the clinical characteristics of the two groups. The average age at diagnosis for the ≤ 40 age group was 34.74 ± 4.94 years; 30 patients were men, and 73 were women. The average age at diagnosis for the > 40 group was 65.50 ± 10.54 years; 32 patients were men, and 68 were women. Among the patients, only 29 had a history of smoking. Significant differences in the largest tumor size were observed between the two groups (*p* < 0.001). Young patients were more likely to have tumors < 1 cm in size, while older patients predominantly had tumors > 2 cm in size. Regarding the location of the tumors, only 2 patients had tumors in both lungs.

**Table 1:** Clinical features of the patients.

Variables	Young (n = 103)	Elder (n = 100)	*p* value
Age (year ± SD)	34.74 ± 4.94	65.50 ± 10.54	< 0.001
Sex, n (%)			0.657
Male	30 (29.13)	32 (32.00)	
Female	73 (70.87)	68 (68.00)	
Smoking history, n (%)			0.059
Never smoker	93 (90.29)	81 (81.00)	
Current or former smoker	10 (9.71)	19 (19.00)	
Largest tumor size			< 0.001
< 1 cm	35 (33.98)	2 (2.00)	*p* < 0.05
1–2 cm	61 (59.22)	48 (48.00)	
> 2 cm	7 (6.80)	50 (50.00)	*p* < 0.05
Tumor location, n (%)			0.744
Ipsilateral	102 (99.03)	99 (99.00)	
Bilateral	1 (0.97)	1 (1.00)	

### Pathological characteristics

The histopathological characteristics of the patients are presented in Table 2. Among the younger patients, the majority were diagnosed with MIA (69.90%), and 29.13% were diagnosed with IAC. Among the older patients, 19.00% were diagnosed with MIA, while 80.00% were diagnosed with IAC. Notably, there was only one case of adenocarcinoma in situ (AIS) in both groups. While young and older patients seemed to have similar subtypes of adenocarcinoma (*p* > 0.05), they had different degrees of differentiation (*p* < 0.001). Compared to young patients, older patients displayed significantly more visceral pleural invasion (4.85% vs. 21.00%, *p* < 0.001), more spread through the air spaces (4.85% vs. 18.00%, *p* < 0.001), and more advanced TNM stages (*p* < 0.001).

**Table 2:** Pathological analysis of lesions.

Characteristics	Young (n = 103), n (%)	Elder (n = 100), n (%)	*p* value
Pathology			< 0.001
AIS	1 (0.97)	1 (1.00)	
MIA	72 (69.90)	19 (19.00)	*p* < 0.05
IAC	30 (29.13)	80 (80.00)	*p* < 0.05
Predominant subtype			0.546
Acinar	12 (40.00)	34 (42.50)	
Papillary	3 (10.00)	9 (11.25)	
Lepidic	14 (46.67)	37 (46.25)	
Solid	1 (3.33)	0 (0)	
Degree of differentiation			< 0.001
Well	5 (16.67)	11 (13.75)	
Well-moderately	12 (40.00)	31 (38.75)	
Moderately	4 (13.3)	33 (41.25)	*p* < 0.05
Moderately poorly	7 (23.3)	2 (2.50)	*p* < 0.05
Poorly	1 (3.33)	0 (0)	
Unknown	1 (3.33)	3 (3.75)	
TNM stage			< 0.001
I A1	57 (55.34)	13 (13.00)	*p* < 0.05
I A2	31 (30.10)	29 (29.00)	
I A3	2 (1.94)	24 (24.00)	*p* < 0.05
I B	4 (3.88)	29 (29.00)	*p* < 0.05
II A	3 (2.91)	3 (3.00)	
II B	2 (1.94)	1 (1.00)	
III A	3 (2.91)	1 (1.00)	
III B	0 (0)	0 (0)	
IV A	1 (0.97)	0 (0)	
VPI	5 (4.85)	21 (21.00)	< 0.001
VTT	2 (1.94)	1 (1.00)	0.551
LVI	4 (3.88)	2 (2.00)	0.355
STAS	5 (4.85)	18 (18.00)	0.003

*AIS* adenocarcinoma in situ, *MIA* minimally invasive adenocarcinoma, *IAC* invasive adenocarcinoma, *VPI* visceral pleural invasion, *VTT* vascular tumor thrombus, *LVI* lymphovascular invasion, *STAS* spread through the airspace.

### Survival analysis

**Figure 1:**
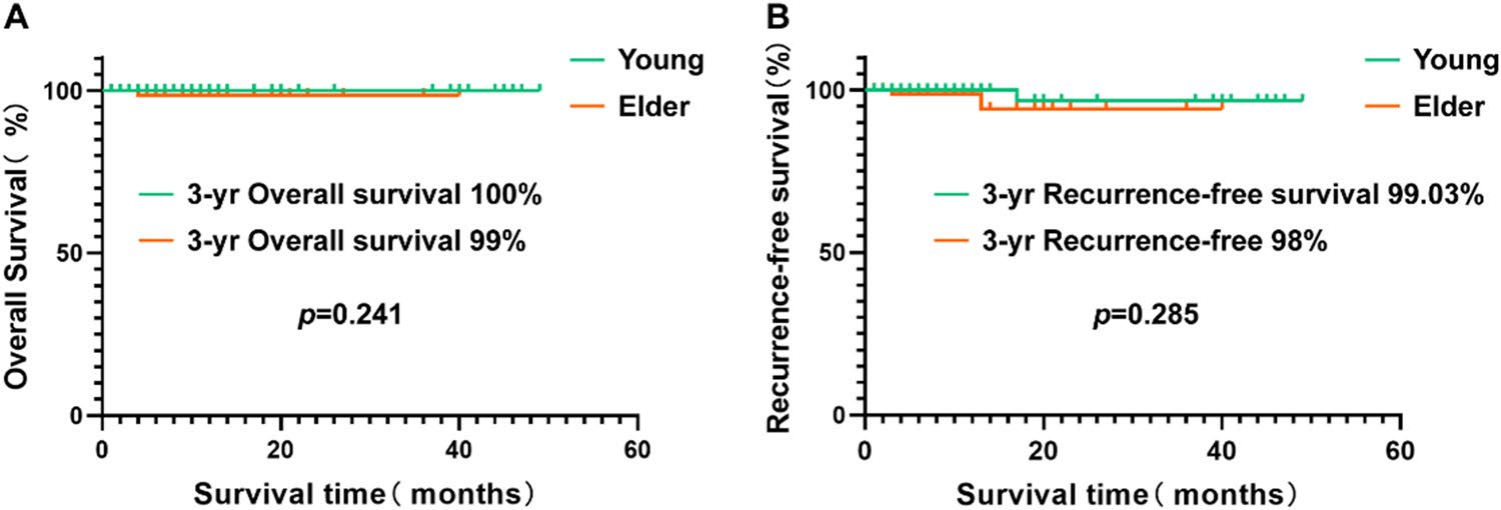
Survival analysis of the two groups of patients. (**A**) 3-year OS of the young group and the elderly group; (**B**) 3-year RFS of the young group and the elderly group.

As of September 2023, all 203 patients had been successfully followed up. A total of 2 patients developed postoperative recurrence, one patient developed distant metastases, and one patient died. One patient had postoperative recurrence in both the young and older groups. One patient with distant metastases and one death were from the older group. Survival analysis revealed that the young group’s 3-year OS and RFS were 100% and 99.03%, respectively, while the 3-year OS and RFS of the older group were 99% and 98%, respectively (Fig. 1).

## Discussion

With improvements in medical technology, the detection of pulmonary ground-glass opacities (GGOs) has increased in recent years^27^. Pulmonary GGOs are more likely in young and older patients^28^. Therefore, it is necessary to define the clinicopathological characteristics and prognosis of young patients with GGO-associated LUAD (GGO-LUAD). In this study, we included 203 patients who underwent VATS and were diagnosed with LUAD between January 2018 and April 2020 due to pulmonary GGO component nodules were include. We divided the patients into younger (≤ 40 years) and older (> 40 years) groups and evaluated the clinical and pathological features of these different groups.

Few GGOs are persistent and malignant; these tumors are more likely to be found in nonsmokers^29,30^. In this study, we found that the proportion of nonsmokers was much more significant than that of smokers among both younger and older patients. These nonsmokers may be affected by risk factors such as environmental pollution and genetic predisposition, resulting in GGOs^31^. The driver genes of young patients diagnosed with LUAD differ from those of older patients^32^. A recent study revealed that young patients have a greater incidence of *ALK* or *EGFR* mutations than older patients^33^. However, in the older group (> 40 years), *KRAS* mutations were found to be the dominant driver mutation^34^.

The size of the GGO is a well‐recognized indicator of malignancy. We found that older patients were more likely to have larger tumors. An increase in size or a solid component is more likely to indicate a malignant tumor^35^. There were more older patients with initial tumors located in the upper and lower lobes of the lungs than younger patients. Most lung cancer cases are located in the upper lobes^36^, and this was observed in both younger and older patients in our study. However, additional studies are needed to explain this phenomenon.

The 3-year OS and RFS of the younger group were 100% and 99.03%, respectively, while the 3-years OS and RFS of the older group were 99% and 98%, respectively. There was no significant difference between the two groups. Specifically, we found that the mean percentage of IAC was more critical in the older group than the young group, indicating that GGOs more easily infiltrated older patients. Although the subtypes of adenocarcinoma in young and older patients seemed to be similar, older patients had more advanced diseases according to tumor-node-metastasis staging. Notably, the proportion of patients with lymph node metastases was low in both groups, consistent with previous research^37–39^.

Our study showed that malignant pulmonary nodules containing GGOs have different pathological subtypes in young patients. Patients with GGOs of different ages have different clinicopathological characteristics. The 3-years prognosis of young patients with malignant pulmonary nodules containing GGOs is satisfactory. Older patients with GGOs need to be closely followed, and timely decision-making should be made according to their condition, as older patients may have a worse clinical prognosis.

We found that GGOs were often suggestive of pulmonary adenocarcinoma and they are easily infiltrated as the ration of MIA and IAC were high in both young and older patients. This means that GGOs needs to be carefully diagnosed and treated. Early surgical treatment is need when the solid component is gradually increasing. Nevertheless, the challenge of mitigating overdiagnosis and overtreatment of GGOs persists. Future research should focus on identifying biomarkers that can effectively differentiate between benign and malignant GGOs. Additionally, given the varying clinical presentations of GGOs in younger and older patients, personalized treatment strategies should be developed to optimize therapeutic outcomes.

This study has several limitations that must be considered. First, it was a retrospective review, meaning intrinsic biases could not be avoided. Second, the study was limited by its single-center nature and small sample size. A multicenter study with a larger sample is needed for further investigation. Additionally, longer follow-ups are necessary to obtain more precise analysis results. Finally, it would be better to apply multivariate analysis to examine the role of different factors in the outcomes of patients with GGOs.

## Data Availability

All data supporting the findings of this study are available within the article.
